# 

**Published:** 1839-06

**Authors:** 


### AMERICAN JOURNAL

OF

### DENTAL SCIENCE,

DEVOTED TO

ORIGINAL ARTICLES,

**REVIEWS OF DENTAL PUBLICATIONS;**

THE LATEST IMPROVEMENTS IN

SURGICAL AND MECHANICAL DENTISTRY,

AND BIOGRAPHICAL SKETCHES OF DISTINGUISHED

DENTISTS.

SURGEON GEN'L'S OFFICE
13369
LIBRARY.

WITH PLATES.

Vol. I.

June 1839 – Sept. 1841.

NEW-YORK.

1839.–41.

WI
AM45M

### ADVERTISEMENT.

The publishers of this Journal have but one great object in view, and that is the dissemination of useful and practical knowledge among Dentists, all of whom are respectfully invited to assist in sustaining the undertaking by becoming subscribers, and also by contributing to the pages of the work. Those who thus desire to co-operate with the publishers, are requested to address their communications to the Secretary of the publishing committee, Solyman Brown, No. 17 Park Place, New-York, inclosing the amount of subscription, viz:—Three Dollars per annum for a single copy, or Two Dollars and Fifty Cents each for any number of copies.

The postage on all letters and communications must be paid by the writers; otherwise they will not be taken from the Post Office.

KELLEY AND FRAETAS,
PRINTERS.

### DENTAL LIBRARY.

The Publishing Committee have the satisfaction to acknowledge the good fortune of having received from Mr. Eleazer Gidney, Dentist, recently from Manchester, England, but formerly of this city, the loan of his Dental Library, consisting of books and prints collected in France and England with much labour and expense. This valuable collection, embracing the principal publications on the subject of the teeth, which have appeared during the last and present century, has been generously placed by Mr. Gidney at the disposal of the publishers of this Journal, subject indeed, to his order at any future day. The catalogue which will be found on the 23d and 24th pages of the present number, it will be seen, does not embrace the works of American writers on the subject; and the publishers respectfully request the authors of Dental Treatises, in the United States, to forward copies to the Publishing Committee in order that a catalogue may be given in our next number. The importance of such a Library as the publishers already possess, augmented by the works of American authors, can hardly be overrated in connexion with the progress of the present undertaking; and the thanks of the Publishing Committee, are hereby respectfully tendered to Mr. Gidney in behalf of the profession generally, for his liberal encouragement of their efforts to establish an American Journal of Dental Science.

*The following is a Catalogue of the Dental Works placed by* E. *GIDNEY, Esq. at the disposal of the Publishing Committee.*

Nat. History of Human Teeth, quarto, London, 1778—John Hunter.
Hostoire Naturelle des Dents. qr.; Paris, 1821—Joseph Fox.
Anatomy of the Teeth, oct.; Philad. 1830—Thomas Bell.
Memoires de la Société Medicale, 8 vo. Paris, 1811—Head of Corvisart.
Première Dentition, 8 vo. Paris, 1806—M. Baumes.
Dental Surgery, 8 vo. London, 1826—Leonard Koecker.
Dentition, 8 vo. London, 1742—Joseph Hurlock.
Structure of the Teeth, 8 vo. Dublin, 1801—Robert Blake.
Practical Directions, 8 vo. London, 1825—Andrew Clark.
New Patent Extractor, 8 vo. London, 1826—J. P. de la Fons.
Parents Dental Guide, 8 vo. London, 1834—William Imrie.
Nat. History of Teeth, 8 vo. London, 1811—Joseph Murphy.
Teeth and Gums, 8 vo. Bath, 1825—M. Sigmond.
Artificial Palates, 8 vo. London, 1824—James Snell.
Diseases of Teeth and Gums, 8 vo. London, 1819—Charles Bew.
Artificial Teeth, 8 vo. London, 1804—N. Dubois De Chemant.
Popular Essay, 8 vo. London, 1815—Richard Downing.
Dentiste de la Jeunesse, 8 vo. Paris, 1817—J. R. Duval.
Dents des Mammifères, 8 vo. Strasbourg, 1823—F. Cuvier.
Seconde Dentition, 8 vo. Paris, 1826—E. M. Miel.
Critical Enquiry, 4 to, London, 1827—George Waite.
Anatomie des Dents, 8 vo. Paris, 1817—H. Serres.
Traité sur les Dents, 8 vo. Paris, 1822—Joseph Lemaire.
Carie de Denti Umani, 8 vo. Genoa, 1812—Francisco Lavagna.
Dents Incorruptibles, 8 vo. Paris, 1821—Joseph Audiban.
Seconde Dentition, 8 vo. Paris, 1826—C. F. Delabarre.
Epitre A. M. Marmont, 8 vo. Paris, 1825—A. Delmond.
Familiar Dissertation, 8 vo. London, 1823—J. P. Hertz.
Extraction des Dents, 12mo. Paris, 1802—J. R. Duval.
Manuel de Dentiste, 12 mo. Paris, 1822.—J. C. F. Maury.
Système Dentaire, 12 mo. Paris, 1820—G. H. Hilaire.
Arrangement des Dents, 12 mo. Paris, 1820—J. R. Duval.
Importance of the Teeth, 12 mo. Philadelphia, 1828—S. S. Fitch.
Mémoire, 12 mo. Paris, 1824—A. Delmond.
Hygiène Dentaire, 12 mo. Paris, 1823—L. E. Dubois.
Dents Incorruptibles, 12 mo. Paris, 1824—Dubois de Chemant.
Le Dentiste Observateur, 12 mo. Paris, An. VI—Mahon.
Maladies Chirurgicales, 12 mo. Paris, 1822—Boyer.
La Seconde Dentition, 12 mo. Paris, 1819—C. F. Delabarre.
La Partie Mechanique, 12 mo. Paris, 1820—C. F Delabarre.
do. do. 2nd tome, 12 mo. Paris, 1820—C. F. Delabarre.
Pathologie, tome 3, 12 mo. Paris, 1822—Joseph Lemaire.
L. Odontotechnie, 12 mo. Paris, 1825—J. Marmont.
La Formation des Dents, 12 mo. Paris, 1766—M. Jourdain.
Dix-sept Articles, 12 mo. Paris, An. VIII ; Louis Laforgue.
Mouth and Teeth, 12 mo. London, 1823 ; J. C. Gerbaux.
Pathologie, tome 2d, 12 mo. Paris, 1824—Joseph Lenaire.
Surgeon Dentist's Manual, 12 mo. London, 1826—G. Waite.
Experiences, &c. 12 mo. Paris, 1746 ; M. Bunon.

Elemens D'Odontalgie, 12 mo. Paris, 1756 ; M. Jourdaine.
Dentiste des Dames, 12 mo. Paris, 1824 ; L. C. Lemaire.
Le Chirurgien Dentiste, 12 mo. Paris, 1746 ; Pierre Fauchard.
Treatise on the Teeth, 12 mo. London ; Barth. Ruspini.
La Beauté, 18 mo. Paris ; L'Ami.
Hygiene de la Bouche, 12 mo. Paris, 1826 ; O. Taveau.
Recherches, &c. 12 mo. Paris, 1757 ; M. Bourdet.
Management of the Teeth, 12 mo. London, 1818 ; L. S. Parmly.
Le Chirurgien Dentiste, 12 mo. Paris, tome 1, 1746 ; Fauchard.
Le Chirurgien Dentiste, 12 mo. Paris, tome 2, 1746 ; Fauchard.
Recherches, &c. 12 mo. Paris, tome 1, 1757 ; Bourdet.
Human Teeth, 12 mo. Utica, 1824 ; E. Gidney.
Treatise, &c. 12 mo. London, 1779 ; Ruspini.
L' Odontotechnie, 12 mo. Paris, 1825 ; Marmont.
Le Chirurgien Dentiste, 12 mo. 12 mo. Paris, tome 1, 1746 ; Fauchard
Formation des Dents, 12 mo. Paris, 1766 ; Jourdain.
Sur le Mercure, 12 mo. Paris, 1811 ; J. S. Vaume.
Obturateur Dentiêr. 12 mo. Paris, 1814 ; M. Fauchard.
Instructions, 12 mo. Paris, 1811 ; J. L. Riviere.
Five Minutes' Advice, 12 mo. London, 1833.
Manuel, &c. 12 mo. Nancy, 1809 ; M. Maggiolo.
Teeth and Gums, 12 mo. London, 1768 ; Thos. Berdmore.
Guide to sound Teeth, 12 mo. New York, 1836 ; S. Spooner.
Doctrina de Morbis Dentium, 12 mo. Vienna, 1778 ; J. J. Plenck.
Anatomie de L'Homme, folio, Paris, 1825 ; Jules Cloquet.
do do quarto, Paris, 1823 ; H. Cloquet.
L'art de conserver des Dents, 18 mo. Paris, 1737 ; M. Gerauldy.

